# Neuronal Chloride Regulation via KCC2 Is Modulated through a GABA_B_ Receptor Protein Complex

**DOI:** 10.1523/JNEUROSCI.2164-16.2017

**Published:** 2017-05-31

**Authors:** Rebecca Wright, Sarah E. Newey, Andrei Ilie, Winnie Wefelmeyer, Joseph V. Raimondo, Rachel Ginham, R.A. Jeffrey Mcllhinney, Colin J. Akerman

**Affiliations:** ^1^Department of Pharmacology and; ^2^Medical Research Council Anatomical Neuropharmacology Unit, University of Oxford, Oxford, OX1 3QT, United Kingdom

**Keywords:** chloride, GABA-B receptor, KCC2, protein complex, synaptic inhibition

## Abstract

GABA_B_ receptors are G-protein-coupled receptors that mediate inhibitory synaptic actions through a series of downstream target proteins. It is increasingly appreciated that the GABA_B_ receptor forms part of larger signaling complexes, which enable the receptor to mediate multiple different effects within neurons. Here we report that GABA_B_ receptors can physically associate with the potassium-chloride cotransporter protein, KCC2, which sets the driving force for the chloride-permeable ionotropic GABA_A_ receptor in mature neurons. Using biochemical, molecular, and functional studies in rodent hippocampus, we show that activation of GABA_B_ receptors results in a decrease in KCC2 function, which is associated with a reduction in the protein at the cell surface. These findings reveal a novel “crosstalk” between the GABA receptor systems, which can be recruited under conditions of high GABA release and which could be important for the regulation of inhibitory synaptic transmission.

**SIGNIFICANCE STATEMENT** Synaptic inhibition in the brain is mediated by ionotropic GABA_A_ receptors (GABA_A_Rs) and metabotropic GABA_B_ receptors (GABA_B_Rs). To fully appreciate the function and regulation of these neurotransmitter receptors, we must understand their interactions with other proteins. We describe a novel association between the GABA_B_R and the potassium-chloride cotransporter protein, KCC2. This association is significant because KCC2 sets the intracellular chloride concentration found in mature neurons and thereby establishes the driving force for the chloride-permeable GABA_A_R. We demonstrate that GABA_B_R activation can regulate KCC2 at the cell surface in a manner that alters intracellular chloride and the reversal potential for the GABA_A_R. Our data therefore support an additional mechanism by which GABA_B_Rs are able to modulate fast synaptic inhibition.

## Introduction

GABAergic synaptic inhibition is mediated by two major receptor systems: ionotropic GABA_A_ receptors (GABA_A_Rs) and metabotropic GABA_B_ receptors (GABA_B_Rs). GABA_A_Rs rely on transmembrane chloride gradients to generate fast inhibitory synaptic currents (Kaila, 1994; Payne et al., 2003). GABA_B_Rs, in contrast, generate slower inhibitory actions via the activation of guanine nucleotide-binding protein (G-protein) signaling pathways (Bettler et al., 2004).

It is becoming increasingly clear that to understand the function and regulation of GABA_B_Rs requires a more complete understanding of the molecular associations that underlie GABA_B_R complexes in the brain. For instance, recent proteomic approaches have identified auxiliary subunit proteins that modulate the receptor's agonist response and kinetics of G-protein signaling (Schwenk et al., 2010). GABA_B_R complexes can also include proteins that are the downstream targets following agonist activation of the receptor (Ciruela et al., 2010b; Park et al., 2010) and proteins that are well placed to control the receptor's dimerization or desensitization (Couve et al., 2001; Pontier et al., 2006). The identification of molecular partners for the GABA_B_R has also revealed a wider range of functions. These include associations that enable GABA_B_R subunits to regulate gene transcription (Nehring et al., 2000; White et al., 2000; Vernon et al., 2001) or the intracellular trafficking of other membrane proteins (Boyer et al., 2009). Further diversity in GABA_B_R function is also likely to relate to the temporal and spatial regulation of the receptor. Recent reports have indicated that the recycling of GABA_B_Rs at the cell surface is dynamic and can be modulated through receptor activation, composition, phosphorylation, or degradation (González-Maeso et al., 2003; Fairfax et al., 2004; Grampp et al., 2007, 2008; Laffray et al., 2007; Vargas et al., 2008; Wilkins et al., 2008; Hannan et al., 2011).

Here we identify and investigate a novel association between postsynaptic GABA_B_Rs and the potassium-chloride cotransporter protein, KCC2. KCC2 contributes to the low intracellular chloride concentrations found in mature neurons and thus establishes the conditions for the hyperpolarizing effect of GABA_A_Rs (Rivera et al., 1999). Furthermore, KCC2 is a locus for modulating the strength of fast synaptic inhibition. Rapid changes in KCC2 function have been shown to be elicited in an activity-dependent fashion and involve different post-translational regulation of the transporter protein, including its phosphorylation state and regulation at the cell surface (Woodin et al., 2003; Rivera et al., 2004; Fiumelli et al., 2005; Lee et al., 2007; Wake et al., 2007; Watanabe et al., 2009; Lee et al., 2010; Chamma et al., 2012; Puskarjov et al., 2012; Medina et al., 2014; Mahadevan and Woodin, 2016).

Using a combination of proteomic, biochemical, and molecular studies, we demonstrate that GABA_B_Rs and KCC2 can functionally associate with one another at the membrane of neurons. Activation of the GABA_B_R results in reduced levels of KCC2 at the cell surface, which parallels an increase in intracellular chloride and depolarizing shift in the reversal potential for the GABA_A_R. Our data support a novel mechanism by which GABA_B_Rs can modulate KCC2 and thereby fast synaptic inhibition mediated by the ionotropic GABA_A_R.

## Materials and Methods

### 

#### 

##### Mass spectrometry.

All experiments using animal tissue were in accordance with regulations from the United Kingdom Home Office Animals (Scientific Procedures) Act. Cortical membranes were prepared by dissecting the cortex from 5 male adult (2 months old) Sprague Dawley rats (Harlan) and homogenizing in 0.32 m sucrose, 50 mm Tris-HCl, pH 7.4 (10 ml/g tissue). The homogenate was centrifuged for 10 min at 690 × *g*_av_, 4°C, and the supernatant centrifuged for 20 min at 8700 × *g*_av_, 4°C. Each pellet was resuspended in 0.32 m sucrose and layered at the top of a sucrose gradient (0.85 m to 1.0 m to 1.2 m sucrose in 50 mm Tris-HCl, pH 7.4). Gradients were centrifuged for 2 h at 111,000 × *g*_av_, 4°C. The membranes were removed, resuspended in 0.32 m sucrose, and centrifuged for 20 min at 19,500 × *g*_av_, 4°C. Each pellet was resuspended in 50 ml of cold dH_2_O with protease inhibitors and placed on ice for 30 min. The samples were centrifuged for 20 min at 34,700 × *g*_av_, 4°C, and the pellets resuspended in 50 mm Tris-HCl, pH 7.4, before determining the protein concentration and freezing at −80°C. For each affinity purification (3 in total), 10 mg of the prepared membranes was solubilized in 50 mm Tris-HCl, pH 7.4, containing 1% sodium deoxycholate, protease inhibitors (Boehringer EDTA free), and 10 mm iodoacetamide. The detergent to protein ratio was 5:1. Lysates were centrifuged for 1 h at 66,700 × *g*_av_, at 4°C. The supernatant was divided equally and rotated with either 5 μg of GABA_B_R1 antibody or sheep IgG for 6 h at 4°C. A 40 μl suspension of Protein G Sepharose Fast Flow beads (GE Healthcare; 1:1 in 50 mm Tris-HCl, pH 7.4) was added and rotated overnight at 4°C. Beads were washed three times with 50 mm Tris-HCl, pH 7.4, + 1% deoxycholate, once with 50 mm Tris-HCl, pH 7.4, and eluted into 25 μl Novex 2× reducing sample buffer by heating at 60°C for 15 min. Samples were analyzed by electrophoresis on 4%–12% Bis-Tris NuPAGE Novex gels with MOPs running buffer (Invitrogen). The gel was stained with GelCode Blue Stain Reagent (Pierce), and bands from both experimental and control lanes were excised. Samples were reduced with dithiothreitol, alkylated with iodoacetamide, and digested with trypsin using a MassPREP workstation (Waters). The resulting peptide mixtures were analyzed by liquid chromatography tandem mass spectrometry (MS/MS) using a CapLC and Q-Tof mass spectrometer (Waters) operating in data-dependent MS/MS mode at the facility at GlaxoSmithKline. Peptides and proteins were identified by automated searching of all MS/MS spectra against a GlaxoSmithKline nonredundant protein database. Candidate proteins associated with the GABA_B_R: (1) had to appear in three independent isolates, (2) be identified at a position on the SDS-PAGE gel that corresponded to their native molecular weight, (3) have been identified on the basis of two or more peptides on each occasion, and (4) not repeatedly appear in IgG control precipitates.

##### Antibodies.

The following antibodies were used in this study: sheep anti-GABA_B_R1 antibody (Pontier et al., 2006), mouse anti-GABA_B_R1 (University of California–Davis/National Institutes of Health NeuroMab Facility, 75-183, clone N93A/49; Sigma, WH0002550M1-100UG, clone 2D7), rabbit anti-GABA_B_R2 (GlaxoSmithKline) (Pontier et al., 2006), sheep IgG (Thermo Fisher Scientific, 31243), rabbit anti-C terminus KCC2 (Millipore, 07-432), rabbit anti-β-tubulin (Sigma, T2200) rabbit anti-GFP (Thermo Fisher Scientific, A11122), rabbit IgG (Thermo Fisher Scientific, 31235), mouse anti-β-tubulin (Cambridge Bioscience, MMS-435P-250), mouse anti-transferrin receptor (Thermo Fisher Scientific, 13-6890), mouse anti-NKCC1 (Developmental Studies Hybridoma Bank, T4 clone), mouse anti-actin (Sigma, A3854), HRP-conjugated donkey anti-rabbit (Stratech, 711-055-152), HRP-conjugated goat anti-mouse (Stratech, 115-055-166), donkey anti-rabbit Cy3-conjugated monovalent Fab fragment (Stratech, 111-167-003), and donkey anti-rabbit Alexa-488-conjugated secondary (Thermo Fisher Scientific, A21206).

##### Preparation of organotypic hippocampal slices.

Electrophysiological recordings, live cell imaging, and biochemistry experiments were conducted in organotypic hippocampal brain slices generated from P7 male Wistar rats and cultured for 7–14 DIV before experimentation. Organotypic hippocampal slices were generated as described previously (Stoppini et al., 1991). Briefly, P7 rat brains were extracted and placed in cold (4°C) Geys Balanced Salt Solution (Sigma), supplemented with d-glucose (34.7 mm). The hemispheres were separated, and individual hippocampi were removed and immediately sectioned into 350-μm-thick slices on a McIlwain tissue chopper. Slices were rinsed in cold dissection media, placed onto Millicell-CM membranes, and maintained in culture media containing 24.5% v/v EBSS, 49% v/v MEM, 24.5% v/v heat-inactivated horse serum, 0.64% w/v glucose, and 2% v/v B27 (all from Invitrogen; 350–360 mOsm) at 36°C in a 5% CO_2_ humidified incubator. The organotypic hippocampal brain slice enabled us to conduct electrophysiological, imaging, and biochemical experiments in the same preparation. A potential source of variance when investigating chloride homeostasis mechanisms in acutely prepared brain slices has been associated with neuronal damage caused during the slicing procedure (Dzhala et al., 2012; Puskarjov et al., 2012). An advantage of the organotypic hippocampal brain slice is that any neurons that are damaged by the slicing process are lost during the culturing period. Indeed, previous work has shown that the pyramidal neurons in the organotypic hippocampal brain slice have mature and stable chloride homeostasis mechanisms, as evidenced by their hyperpolarizing E_GABAA_ (Ilie et al., 2012; Raimondo et al., 2012; Ellender et al., 2014). This is supported by the current work, which observed that E_GABAA_ is affected by KCC2-blocking drugs, but not by NKCC1-blocking drugs (see below). At the time of electrophysiological recording (P7 + 7–14 DIV), CA3 pyramidal neurons in the organotypic hippocampal slices exhibited a hyperpolarizing E_GABAA_ (−82.8 ± 1.4 mV) compared with their resting membrane potential (−71.5 ± 0.9 mV, *n* = 13; *p* < 0.001), and their E_GABAA_ shifted to more depolarized values upon application of 1 mm furosemide (E_GABAA_ in furosemide = −70.2 ± 2.9 mV; *n* = 12) or 25 μm VU0240551 (E_GABAA_ in VU0240551 = −75.8 ± 2.9 mV) (Delpire et al., 2009). This is consistent with KCC2 being active in these neurons and contributing to a mature and hyperpolarizing E_GABAA_. Although many aspects of organotypic hippocampal slice cultures have been shown to resemble the *in vivo* state (De Simoni et al., 2003), excitatory neurons in this experimental system exhibit increased axonal sprouting, which is likely to underlie the higher levels of synchronous network activity (Dyhrfjeld-Johnsen et al., 2010).

##### Heterologous cell culture and transfection of KCC2 constructs.

CHO cells stably expressing GABA_B_R1a/R2 or GABA_B_R1b/R2 were grown as described previously (Pontier et al., 2006) in DMEM/F12 Ham (Invitrogen) with 2 mm glutamine (Invitrogen), 10% v/v FBS (Invitrogen), 0.5 mg/ml geneticin, 0.4 mg/ml hygromycin B, and 2.5 μg/ml puromycin (all from Invitrogen; pH 6.8–7.2; 290–330 mOsm). Full-length rat KCC2 cDNA sequence encoding amino acids 1–1116, as well as KCC2 deletion mutants transmembrane domain (TMD) + carboxy-terminal domain (CTD) (amino acids 97–116), amino-terminal domain (NTD) + TMD (amino acids 1–640), TMD (amino acids 97–640), and CTD (amino acids 637–1116), were cloned into pEGFP-N3 (Clontech) to generate C-terminally tagged fusion proteins. KCC2 NTD (amino acids 1–100) was cloned into pEGFP-C3. Constructs were transfected into CHO cells using JetPEI (Polyplus) and expressed for 48 h before immunoprecipitation or biotinylation analysis (see below).

##### Coimmunoprecipitation.

Organotypic hippocampal slices or transfected CHO cells were homogenized in CHAPS buffer (50 mm Tris, pH 7.5, 150 mm NaCl, 5 mm EDTA, 0.5% w/v CHAPS, and protease inhibitors; Roche). Precleared lysates were probed for GABA_B_R1, KCC2, GFP, or IgG. Protein A/G + agarose was added for 2 h before washing in CHAPS buffer. Agarose beads were eluted in 2× sample buffer at 60°C for 10 min, before loading on to 6% or 8% SDS-PAGE gels. Gels were immunoblotted onto Protran nitrocellulose membranes (Sigma) and probed with indicated primary antibodies overnight at 4°C, before addition of relevant secondary HRP-conjugated antibodies and development with Pierce ECL substrate (Thermo Fisher Scientific).

##### Biotinylation of cell surface proteins.

Rat organotypic hippocampal slices were incubated for 20 min at 28°C–30°C in either control ACSF or ACSF containing 5 μm SKF97541 while continuously bubbling with 95% O_2_-5% CO_2_. For biotinylation of both slices and CHO cells, every subsequent step was performed on ice. Samples were incubated for 30–45 min with 100 μm cleavable biotin (EZ-Link Sulfo-NHS-SS-Biotin, Thermo Fisher Scientific), then washed twice with 100 μm lysine and lysed with lysis buffer (20 mm Tris, pH 7.5, 50 mm NaCl, 1 mm EDTA, 0.1% w/v SDS, 1% v/v Triton X-100 containing protease inhibitors; Roche). The lysate was centrifuged, and 50 μl of the resultant supernatant was removed as the “total” protein lysate sample. Biotinylated proteins were captured by incubation with washed NeutrAvidin Ultralink Resin (Thermo Fisher Scientific) on a rotator overnight at 4°C. The beads were washed 3 × with lysis buffer and the “Surface” sample eluted at 37°C for 30 min in 2× sample buffer. Prepared protein samples were subjected to SDS-PAGE/immunoblotting, as described above. In the CHO cell experiments, fluorescent signals were analyzed using a LI-COR Odyssey scanner. For slice experiments, the ECL signal was captured digitally using a Fluor-S MultiImager (Bio-Rad). Background intensity was subtracted and the optical density for each band quantified through Quantity One version 4.1.0 software (Bio-Rad).

For biotinylation experiments in organotypic hippocampal slices, each sample was comprised of 3 slices from the same animal, maintained on the same Millicell-CM membrane. Every SKF97541-treated sample was processed in parallel with a control sample from the same animal. Between 2 and 8 samples were generated from an individual animal, and each experimental drug manipulation used tissue from between 2 and 6 animals. For each sample, the surface protein was normalized against the total protein, which was run in the adjacent lane. As the ratio of surface/total was calculated within each sample, this controlled for differences in overall protein levels across samples and variance associated with loading. “Control” and corresponding “SKF97541-treated” samples were always run on the same gel; control values were set to 100% and the SKF97541 treatment expressed as a percentage of control. If the surface/total ratio for a particular protein was consistently lower for SKF97451-treated samples than their corresponding control samples, this would result in a population mean <100% and would indicate that GABA_B_R activation caused a decrease in surface levels of the protein.

##### Immunofluorescence.

Organotypic hippocampal slices (P7 + 7–14 DIV) were fixed either in ice-cold methanol (for KCC2 labeling) or in 4% PFA followed by cold methanol (for GABA_B_R2 labeling). Slices were blocked in PBS containing 0.3% Triton X-100 and 5% normal goat serum. Incubation with primary antibodies (1/1000 dilution for both rabbit anti-GABA_B_R2 and rabbit anti-KCC2) was performed at 4°C overnight. Slices were washed 4 times with PBS containing 0.3% Triton X-100 and incubated for 4 h at room temperature in the same buffer supplemented with 5% normal goat serum and containing either a 568- or 488-coupled anti-rabbit secondary antibody (Invitrogen). Slices were then washed a further 4 times before mounting in 50% glycerol/PBS.

Immunofluorescence was also examined in dissociated hippocampal neurons, where antibody and optical access is better, and where we were able to develop a protocol to quantify coexpression of both proteins within the same cell. Rat dissociated hippocampal cells were prepared at embryonic day 18 (E18) as described previously (Pooler et al., 2009). After 18–21 DIV cells were fixed and permeabilized in ice-cold methanol, blocked in donkey serum, and sequentially colabeled for GABA_B_R2 and either KCC2 or β-tubulin. Cells were incubated with the rabbit anti-GABA_B_R2 primary antibody (1/200 dilution), followed by incubation with a donkey anti-rabbit Cy3-monovalent Fab fragment (1:250 dilution). After washing, the cells were further colabeled for KCC2 (1/1000) or β-tubulin (1/500; both anti-rabbit) followed by incubation with donkey anti-rabbit Alexa-488-conjugated secondary antibody. Appropriate controls were performed to ensure that the Cy3 Fab fragment blocked all available GABA_B_R sites. Coverslips were washed and mounted with Vectashield mounting medium (Vector Laboratories). Images were collected using a Zeiss Plan-Apochromat 63×, 1.4 NA oil objective, mounted on a Zeiss LSM510 confocal scanning microscope, mounted on an Axiovert 100M inverted microscope (Carl Zeiss). Hippocampal neurons in the dissociated cultures were identified as having a large soma and dendritic spines.

##### Electrophysiological recordings.

Organotypic hippocampal slices were transferred to a recording chamber and continuously superfused with 95% O_2_/5% CO_2_ ACSF, heated to 28°C–30°C. These conditions ensured thermal stability and permitted long-term patch-clamp recordings from CA3 pyramidal neurons. The ACSF was composed of the following (in mm): 120 NaCl, 3 KCl, 2 MgCl_2_, 2 CaCl_2_, 1.2 NaH_2_PO_4_, 23 NaHCO_3_, 11 d-glucose, pH 7.3–7.4. With the exception of the synaptic stimulation experiments, the ACSF also contained 1 μm TTX (Tocris Bioscience) to eliminate any potential effects at the network level. For perforated patch recordings, the internal solution contained the following (in mm): 135 KCl, 4 Na_2_ATP, 0.3 Na_3_GTP, 2 MgCl_2_, and 10 HEPES, osmolarity 290 mOsm, pH 7.35. Gramicidin (Calbiochem) was added on the day of the experiment to achieve a final concentration of 80 μg/ml. Recordings were made with 2–4 mΩ pipettes via an Axopatch 1D amplifier (Molecular Devices), once perforation had reached a steady access resistance of between 20 and 60 mΩ. To measure E_GABAA_, cells were maintained at a holding potential of −60 mV, from which they received voltage steps ranging from −30 to −90 mV. Reported membrane potentials were corrected for the voltage drop across the series resistance for each neuron. The liquid junction potential associated with the perforated-patch recordings was small (2.7 mV), and so membrane potential values were not adjusted for this parameter. GABA_A_R activation was achieved by pressure application of muscimol (10 μm, Tocris Bioscience) via a picospritzer (General Valve). To minimize errors associated with access resistance, muscimol-evoked currents were kept small (corresponding to conductances of up to 40 nS) by adjusting the position of the muscimol pipette. Each voltage step lasted for 8000 ms, and cells were returned to the resting holding potential of −60 mV for 30 s between steps to allow time for the intracellular chloride to reequilibrate (Ehrlich et al., 1999). As a further precaution, the direction in which these voltage steps progressed (i.e., from −30 to −90 mV, or from −90 to −30 mV) was alternated to avoid any bias in the E_GABAA_ calculations brought about by chloride loading or removal (Akerman and Cline, 2006). Consistent with the fact that transient changes in E_GABAA_ caused by transmembrane fluxes of chloride recover with a time constant of ∼15 s (Raimondo et al., 2012), our protocol produced reliable estimates of steady-state E_GABAA_. All drugs were added to the ACSF, with the exception of pertussis toxin (PTX, Sigma) and okadaic acid (Tocris Bioscience), which were added directly to the tissue culture media before experimentation. As required, the following antagonists and blockers were added to the external bathing solution; SKF7541, CGP55845, SCH23390, K252a, d-AP5, kynurenic acid, SR95531, and VU0240551 (all from Tocris Bioscience), furosemide, bumetanide, Gö6976, sodium orthovanodate (Na_3_VO_4_), and nimodipine, thapsigargin, and monodansylcadaverine (DC) (all from Sigma).

For synaptic stimulation experiments, glutamatergic transmission was blocked by adding 2 mm kynurenic acid to the ACSF, and GABA release at synaptic terminals was evoked by delivering electrical stimuli via a bipolar tungsten stimulating electrode (FHC), placed 50–100 μm from the recorded pyramidal cell, at the border of the stratum pyramidale and stratum radiatum (Scanziani, 2000). To establish the stimulation conditions under which synaptic GABA_A_R and GABA_B_R responses are evoked, a series of recordings were first performed in whole-cell mode using a low-chloride internal solution containing the following (in mm): 140 K-gluconate, 2 Na_2_ATP, 3 Na_3_GTP, 2 MgCl_2_, 1 EGTA, and 5 HEPES. To improve detection of GABAergic currents in the whole-cell recordings, cells were clamped at −50 mV, and GABA_A_R and GABA_B_R conductances were calculated by dividing the isolated currents by their driving force (see Fig. 7*C*).

Synaptic E_GABAA_ was determined from gramicidin perforated patch recordings using a step voltage-protocol from a holding potential of −60 mV. The holding potential of the cells was stepped at 5 mV increments between −60 and −90 mV, during which pure GABA_A_R currents were elicited using single presynaptic stimuli (see Fig. 7). Again, 30 s was allowed between presynaptic stimuli to allow time for the intracellular chloride to reequilibrate (Ehrlich et al., 1999). In synaptic conditioning experiments, a stimulation protocol was used to strongly activate GABA_B_Rs (bursts of 6 stimuli at a frequency of 20 Hz, repeated every 5 s for a period of 75 s; see Fig. 7) and the effects upon synaptic E_GABAA_ were measured. During the GABA_B_R synaptic conditioning protocol, the postsynaptic neuron was held at its E_GABAA_ to avoid transient loading of the cells with chloride (see Fig. 7*B*). In addition, to allow time for any transient changes to intracellular chloride to fully reequilibrate (Raimondo et al., 2012; Ehrlich et al., 1999), the first measurement of synaptic E_GABAA_ following the GABA_B_R stimulation protocol was made after 5 min. As before, synaptic E_GABAA_ was measured using single presynaptic stimuli to activate pure GABA_A_R currents.

Wherever possible, electrophysiological recordings were conducted under a “within cell” experimental design. This means that each neuron had E_GABAA_ measurements before (“baseline”) and after drug treatment, so that each neuron served as its own control. This “within-cell” experimental design reduces the impact of cross cell variability, means that drug effects can be expressed as a change in E_GABAA_, and also means that effects can be examined using paired statistical tests. For clarity, we report the mean absolute values of E_GABAA_ and the mean change in E_GABAA_. To minimize the potential effect of changes in recording conditions that may have taken place over the course of the study, we took the additional step of restricting comparisons to recordings that were performed during similar time periods. For example, over the course of the study, the effect of SKF97541 upon E_GABAA_ was measured in a total of 20 neurons. However, when compared with another experimental group, the SKF97541 data were restricted to recordings performed during a similar time period as the experimental group. Finally, to avoid potential contamination effects across experiments, only one electrophysiological recording was performed per organotypic hippocampal brain slice. This meant that, for each experiment, the number of neurons corresponds to the number of slices. The slices for an individual experiment were generated from between 4 and 10 animals, depending on the particular sample size and complexity of the experiment.

##### Measurements of intracellular chloride with the Cl-sensor protein.

The cyan and yellow fluorescent protein (CFP-YFP) based ratiometric Cl-Sensor protein (Markova et al., 2008) was delivered to CA3 pyramidal neurons in organotypic hippocampal slice cultures by biolistic transfection (Bio-Rad). At 2–3 d after transfection, Cl-Sensor protein expressing neurons were imaged using an FV300 confocal microscope (Olympus), custom-converted for multiphoton imaging, and equipped with a MaiTai-HP Ti:sapphire femtosecond pulsed laser (Newport Spectra-Physics). Images were acquired using Fluoview software (version 5.0, Olympus). Cells were excited at 850 nm and a 510 nm dichroic mirror was used to separate emitted light into CFP and YFP channels, which were filtered at 460–500 nm and 520–550 nm, respectively, and detected simultaneously using two externally mounted PMTs (Hamamatsu). Image stacks were flat-field corrected, collapsed along the *z*-plane, background subtracted, and the YFP/CFP ratio was calculated by dividing the respective images on a pixel-by-pixel basis. The ratio was calibrated to absolute intracellular chloride values using the K^+^/H^+^ exchanger nigericin and the Cl^−^/OH^−^ exchanger tributyltinchloride (both at 20 μm) in a high K^+^, HEPES-buffered solution at pH 7.35, as described previously (Boyarsky et al., 1988; Kuner and Augustine, 2000).

##### Data analysis and statistics.

Data and statistical analyses were performed using MATLAB R2008b (The MathWorks) and SPSS (IBM). All data are reported as mean ± SE. Statistical comparisons were made using either paired or unpaired Student's *t* tests, and one-way ANOVAs with *post hoc* Dunnett (two-sided) corrections. All statistical tests were two-tailed, and a *p* value of <0.05 was deemed statistically significant.

## Results

### GABA_B_Rs form a protein complex with the potassium-chloride transporter KCC2 at the neuronal membrane

**Figure 1.**
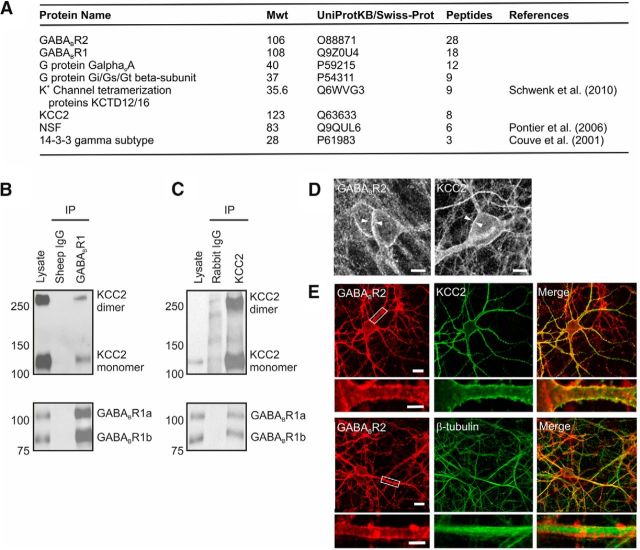
GABA_B_Rs associate with KCC2 at the cell membrane in cortex and hippocampus. ***A***, Affinity purification and mass spectrometry were used to identify proteins associated with the GABA_B_R in synaptic membrane preparations from adult rat cortex. The table shows the number of unique peptides for each protein, obtained across three independent isolates of the GABA_B_R1 protein. Where the identified proteins have previously been shown to associate with the GABA_B_R, references are provided. ***B***, KCC2 coimmunoprecipitates with the GABA_B_R. Rat hippocampal lysates were immunoprecipitated with an anti-GABA_B_R1 antibody, and subsequent Western blot analysis for KCC2 revealed two distinct bands at ∼130 and 270 kDa, which correspond to the monomeric and dimeric forms of KCC2 (top blot, right lane). Probing for GABA_B_R1 confirmed successful immunoprecipitation of both the GABA_B_R1a and GABA_B_R1b isoforms (bottom blot). In contrast, controls using sheep anti-IgG antibody failed to pull down KCC2 or GABA_B_R1 (middle lanes). ***C***, The GABA_B_R coimmunoprecipitates with KCC2. Immunoprecipitates from rat hippocampal lysates isolated with an anti-KCC2 antibody were positive for GABA_B_R1a, GABA_B_R1b, and KCC2 (top and bottom blots, right lanes). In contrast, control experiments using a rabbit anti-IgG failed to pull down KCC2 or GABA_B_R1 from the same lysate (middle lanes). ***D***, The GABA_B_R (left) and KCC2 (right) are both localized at the plasma membrane of pyramidal neurons, as revealed by rabbit polyclonal antibodies against GABA_B_R2 (left) and KCC2 (right) in separate rat organotypic hippocampal slices (P7 + 7–14 DIV). Scale bars, 10 μm. ***E***, Using a sequential double-labeling technique in dissociated neuronal cultures (see Materials and Methods), GABA_B_R2 (red) and KCC2 (green) were found to be colocalized (yellow) at somatic and dendritic membranes of hippocampal pyramidal neurons. Magnifications of the areas highlighted within the white boxes are provided in the panels below. Control staining (bottom) for GABA_B_R2 and β-tubulin revealed nonoverlapping signals. Scale bars, 20 and 5 μm.

We used a combination of coimmunoprecipitation and mass spectrometry to identify functionally important components of GABA_B_R protein complexes in the brain. An anti-GABA_B_R1 antibody was used to isolate antibody-protein complexes from membrane preparations generated from freshly dissected adult rat cortex (see Materials and Methods). Analysis of the resulting peptides revealed a series of proteins that have previously been shown to associate with the GABA_B_R, including G-protein subunits (Bettler et al., 2004), potassium channel tetramerization proteins (Schwenk et al., 2010), NEM-sensitive fusion protein (Pontier et al., 2006), and 14-3-3 signaling proteins (Couve et al., 2001) (Fig. 1*A*). In addition, the mass spectrometry revealed a novel potential association between the GABA_B_R and the solute carrier family 12, member 5 protein (SLC12A5), also known as the potassium-chloride cotransporter KCC2 (Fig. 1*A*). As with all of the associated proteins, KCC2 was present in three independent neuronal membrane isolates, where it was identified from multiple peptides on each occasion and did not appear in IgG control precipitates (Fig. 1*B*).

KCC2 coimmunoprecipitated robustly with GABA_B_R1 when protein complexes were isolated from rat organotypic hippocampal brain slices using either a GABA_B_R1 (Fig. 1*B*) or a KCC2 antibody (Fig. 1*C*). KCC2 coimmunoprecipitated with the two splice isoforms of the GABA_B_R1 subunit: GABA_B_R1a and GABA_B_R1b (Fig. 1*C*). KCC2 appeared as two bands (130 and 270 kDa) on immunoblots (Fig. 1*B*,*C*), consistent with previous reports that KCC2 can exist as both a monomer and a dimer (Blaesse et al., 2006; Uvarov et al., 2009). This confirmed that the association between GABA_B_R and KCC2 is present in organotypic hippocampal rat brain slices as well as acutely dissected rat cortex, and revealed that the GABA_B_R associates with both monomeric and dimeric forms of KCC2, although it is not clear whether this is a direct interaction or whether other proteins are involved. Consistent with the biochemical evidence, immunofluorescence staining in rat organotypic hippocampal slices (P7 + 7–14 DIV) and rat dissociated hippocampal cultures (E18 + 18–21 DIV) confirmed that the GABA_B_R and KCC2 are both found at somatic and dendritic membranes and exhibit overlapping labeling (Fig. 1*D*,*E*). The immunofluorescence protocol in dissociated cultures enabled us to examine coexpression of both proteins within the same cell (see Materials and Methods; Fig. 1*E*). We therefore quantified the GABA_B_R and KCC2 staining pattern in the dissociated hippocampal neurons and found that the vast majority exhibited overlapping labeling at the membrane, consistent with colocalization of the proteins (91%; 52 of 57 GABA_B_R-positive cells).

**Figure 2.**
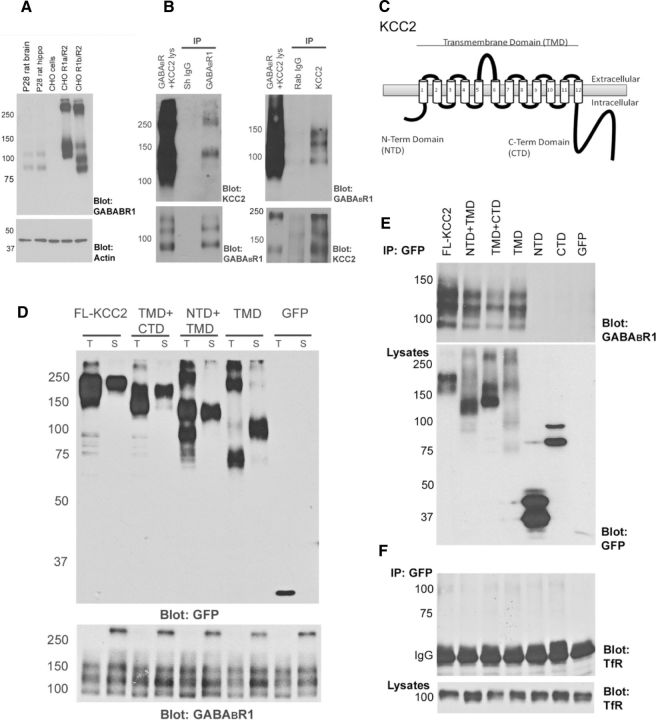
The GABA_B_R associates with the transmembrane domain of KCC2. ***A***, The GABA_B_R1 monoclonal antibody 2D7 used for immunoblotting is specific for GABA_B_R1. In Western blots of total rat brain and rat hippocampus homogenate, two bands corresponding to GABA_B_R1a and GABA_B_R1b were detected. Blots of lysates from CHO cells stably expressing either GABA_B_R1a/R2 or GABA_B_R1b/R2 revealed multiple bands, consistent with differential glycosylation of GABA_B_R1 proteins in this system. ***B***, The KCC2-GABA_B_R association can be reconstituted in a heterologous cell system. CHO cells stably expressing GABA_B_R1b/R2 were transiently transfected with FL-KCC2-GFP and used for coimmunoprecipitation experiments. Western blots of resulting complexes showed that KCC2 can be coimmunoprecipitated with GABA_B_R1b (left), and the reciprocal coimmunoprecipitation confirms the association (right). IgG controls were included in each experiment. Sh, Sheep; Rab, rabbit. ***C***, Schematic diagram of KCC2 showing the intracellular NTD, CTD, and TMD with its 12 predicted transmembrane helices. ***D***, GFP fusion proteins of FL-KCC2 and different KCC2 deletion constructs containing the TMD are expressed and transported to the plasma membrane. Biotinylation experiment comparing total (T) and cell surface (S) protein levels in CHO GABA_B_R1b/R2 cells, transiently transfected with different GFP fusion constructs (left). FL-KCC2 (predicted molecular mass 150 kDa, although the TM region is glycosylated), TMD + CTD (predicted 140 kDa), NTD + TMD (predicted 96 kDa), and TMD (predicted 86 kDa) are all detected on the cell surface, whereas GFP (27 kDa) alone is not. Additional bands detected likely represent alternatively glycosylated, degraded, or aggregated proteins. The blot was reprobed with GABA_B_R1 antibody to confirm surface expression of the receptor (bottom). ***E***, The transmembrane domain of KCC2 is required for the association with the GABA_B_R. Coimmunoprecipitation experiments on CHO GABA_B_R1b/R2 cells transiently expressing KCC2-GFP deletion constructs were performed using anti-GFP as the precipitating antibody. When the resulting complexes were probed for GABA_B_R1 (top), all KCC2-GFP fusion proteins containing the TMD successfully coimmunoprecipitated GABA_B_R1b. However, GFP fusion proteins containing only the NTD (predicted 38 kDa) or the CTD (predicted 82 kDa) did not capture GABA_B_R1b. Under these conditions, and compared with FL-KCC2 (100%), the relative amounts of GABA_B_R detected after isolation with the GFP antibody was 68 ± 16% for NTD + TMD, 36 ± 2% for TMD + CTD, 76 ± 19% for TMD, 3 ± 1% for NTD, 3 ± 2% for CTD, and 3 ± 1% for GFP (*n* = 2–4 in each case). These quantifications will be influenced by the level of expression of each of the different constructs. Cell lysates expressing the GFP fusion proteins are shown (bottom). Additional bands detected with the GFP antibody are likely to be alternatively glycosylated, degraded, or higher-order aggregates of the expressed fusion proteins. ***F***, Control experiments show that the endogenous 100 kDa transferrin receptor (TfR) is not coimmunoprecipitated with KCC2-GFP proteins in CHO GABA_B_R cells (top; IgG bands are shown for clarity). Lysates are also shown (bottom).

To further characterize the association between GABA_B_Rs and KCC2, we used a heterologous CHO cell line that constitutively expresses rat GABA_B_R1b and GABA_B_R2 (CHO GABA_B_R1b/R2) (Pontier et al., 2006). GABA_B_R1b from this cell line was detected as multiple bands on immunoblots, consistent with differential glycosylation of the GABA_B_R1b protein in this system (Fig. 2*A*). CHO GABA_B_R1b/R2 was transfected with recombinant versions of rat KCC2 fused to GFP. Coimmunoprecipitation experiments using antibodies against GABA_B_R1, KCC2 (Fig. 2*B*), or GFP (Fig. 2*E*) confirmed that the association between GABA_B_Rs and full-length KCC2 (FL-KCC2) could be reconstituted in this system. KCC2 is predicted to consist of a cytoplasmic amino-terminal domain and a cytoplasmic carboxy-terminal domain, either side of a transmembrane domain that contains 12 transmembrane helices (Fig. 2*C*) (Payne et al., 1996). We generated GFP fusions of KCC2 that were comprised of only the NTD, only the CTD, or only the TMD. In addition, we generated GFP fusions of KCC2 that lacked either the amino-terminal domain (TMD + CTD) or the carboxy-terminal domain (NTD + TMD). Biotinylation experiments confirmed that each of the fusion proteins containing the transmembrane domain was trafficked, at least in part, to the cell surface in CHO cells (Fig. 2*D*). This is consistent with previous studies that have shown that KCC cytoplasmic domains (N and C terminal) are not essential for membrane delivery in heterologous cell systems (Casula et al., 2001; Li et al., 2007). We then performed coimmunoprecipitation experiments to establish the KCC2 region responsible for the association with GABA_B_Rs. These revealed that GABA_B_Rs can form a complex with versions of KCC2 that lack both intracellular terminal domains. However, GABA_B_Rs do not associate with versions of KCC2 that lack the transmembrane domain (Fig. 2*E*). The association appears specific as another transmembrane protein, the transferrin receptor, was not found in the KCC2 isolates (Fig. 2*F*). Thus, KCC2 associates with the GABA_B_R via its transmembrane domain, which is consistent with the idea that KCC2 and GABA_B_R can form a protein complex at the neuronal membrane.

### GABA_B_R activation affects transmembrane chloride gradients

Signaling interactions across GABA_B_R protein complexes have been shown to be capable of modulating the activity of both the receptor and its associated proteins (Balasubramanian et al., 2004; Pontier et al., 2006; Ciruela et al., 2010b; Park et al., 2010). Given the evidence that the GABA_B_R and KCC2 can associate at the membrane, we investigated whether activation of the GABA_B_R can influence how KCC2 contributes to transmembrane chloride gradients. To assess KCC2 function, intracellular chloride concentration ([Cl^−^]_i_) was monitored by calculating the reversal potential of the ionotropic GABA_A_R (E_GABAA_). To avoid disrupting [Cl^−^]_i_, gramicidin perforated patch-clamp recordings were conducted from CA3 pyramidal neurons in rat organotypic hippocampal slices (P7 + 7–14 DIV). Neurons were clamped at a series of membrane potentials, and GABA_A_R currents were evoked by delivering brief puffs of the selective GABA_A_R agonist muscimol (10 μm) onto the cell soma. The mean resting membrane potential was −71.5 ± 0.9 mV, compared with an E_GABAA_ value of −82.5 ± 1.4 mV (*n* = 13). Thus, the neurons displayed a mature hyperpolarizing E_GABAA_ profile at the time of recording, consistent with KCC2 expression and function.

**Figure 3.**
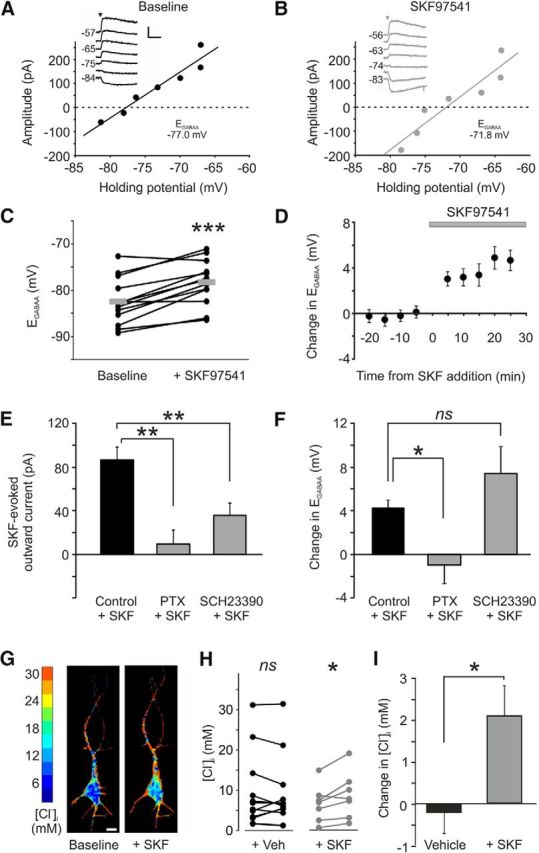
GABA_B_R activation causes a depolarizing shift in E_GABAA_ and increase in intracellular chloride. ***A***, Example GABA_A_R *I–V* plot from a gramicidin perforated patch recording of a CA3 pyramidal neuron in a rat organotypic hippocampal slice. Inset, Raw current traces recorded at different holding potentials. GABA_A_R currents were evoked by local application of muscimol (10 μm, arrowhead) to the cell soma. E_GABAA_ was defined as the holding potential at which the GABA_A_R current had an amplitude of zero. Calibration: 100 pA, 1 s. ***B***, Example *I–V* plot from the same cell in ***A*** recorded after 10 min of bath application of the GABA_B_R agonist, SKF97541 (1 μm). ***C***, GABA_B_R activation led to a significant depolarizing shift in E_GABAA_ across a population of neurons (*n* = 13). ***p = 0.0009 (paired *t* test). Each connected pair of dots corresponds to an individual neuron. Gray horizontal bars represent population means. ***D***, Change in E_GABAA_ plotted as a function of time before and after the onset of SKF97541 application (gray bar; *n* = 13). ***E***, Disrupting G-protein signaling with PTX or blocking GIRK channels with SCH23390 resulted in a significant reduction in the SKF97541-evoked current compared with control neurons (*n* = 7, *n* = 8, and *n* = 13, respectively). **p ≤ 0.01 (ANOVA followed by *post hoc* Dunnett's correction). ***F***, PTX (*n* = 7) significantly reduced the shift in E_GABAA_ following GABA_B_R activation compared with control cells (*n* = 13). *p = 0.03 (ANOVA followed by *post hoc* Dunnett's correction). In contrast, blocking GIRK channels did not prevent the shift in E_GABAA_ (*n* = 7). *p* = 0.24 (ANOVA followed by *post hoc* Dunnett's correction). ***G***, Chloride concentration images of a CA3 pyramidal neuron expressing the Cl-Sensor protein and recorded before (left) and after (right) bath application of SKF97541. Scale bar, 10 μm. ***H***, Although there was no change in chloride concentration in control neurons expressing the Cl-Sensor protein and exposed to vehicle for 20 min (left; +Veh, *p* = 0.67, paired *t* test, *n* = 13), activation of GABA_B_Rs with SKF97541 (5 μm for 20 min) resulted in a significant increase in [Cl^−^]_i_ (right;+SKF, *p = 0.03, paired *t* test, *n* = 7). ***I***, Activation of GABA_B_Rs caused a significant increase in [Cl^−^]_i_ after 20 min compared with control cells imaged over the same time period (*p = 0.02, *t* test).

To investigate whether agonist activation of GABA_B_Rs mediates functional changes in [Cl^−^]_i_, E_GABAA_ was measured before and after the application of the specific GABA_B_R agonist, SKF97541 (1 μm). GABA_B_R activation was found to result in a depolarizing shift in E_GABAA_, which was evident 5–10 min following GABA_B_R activation and persisted for the remainder of the recording (Fig. 3*A–D*). Across a population of CA3 pyramidal neurons, the mean E_GABAA_ shifted from a baseline value of −82.5 ± 1.4 mV to −78.2 ± 1.3 mV following GABA_B_R activation. This represented a mean change in E_GABAA_ of 4.2 ± 0.7 mV (*p* = 0.0009, *n* = 13, paired *t* test; Fig. 3*D*). In control experiments, blocking GABA_B_Rs with a selective, competitive antagonist (5 μm CGP55845) prevented the change in E_GABAA_ in response to SKF97541 (*p* = 0.2, *n* = 6, paired *t* test). This confirmed that the effects were specific to the GABA_B_R, and not alternative receptors such as the GABA_C_R.

Further experiments established that the GABA_B_R-mediated effect requires associated G-proteins, but is independent of downstream, G-protein-coupled inwardly rectifying potassium (GIRK) channels. First, G-protein signaling via the GABA_B_R was disrupted by pretreating the organotypic hippocampal slices with the G_i_/_0_-protein antagonist PTX (5 μg/ml for 24 h before recordings). We could confirm that PTX treatment did disrupt GABA_B_R G-protein-coupled signaling because the SKF97541-evoked membrane current that is associated with GIRK channel activity was significantly smaller in PTX-treated neurons (9.4 ± 12.8 pA at a holding potential of −60 mV, *n* = 7) than in control neurons (86.0 ± 11.4 pA, *n* = 13, *p* = 0.0003, ANOVA followed by *post hoc* Dunnett's correction; Fig. 3*E*). Importantly, when we examined the SKF97541-induced effect upon E_GABAA_, we found that this was significantly inhibited after PTX treatment. The mean E_GABAA_ baseline for PTX-treated cells was −75.6 ± 3.1 mV and showed little change following SKF97541 treatment when it was −76.6 ± 3.1 mV. This was significantly different to the effect observed in control cells (*p* = 0.034, *n* = 13 and *n* = 7, respectively, ANOVA followed by *post hoc* Dunnett's correction; Fig. 3*F*). In contrast, using SCH23390 (10 μm) (Kuzhikandathil and Oxford, 2002) to block the downstream GIRK channels directly, did not prevent the SKF97541-induced change in E_GABAA_. The efficacy of the GIRK channel block by SCH23390 was evident from the significant reduction in the SKF97541-evoked current (35.5 ± 11.3 pA in SCH23390-treated neurons compared with 86.0 ± 11.4 pA in control neurons, *n* = 8 and 13, respectively, *p* = 0.01, ANOVA followed by *post hoc* Dunnett's correction; Fig. 3*E*). However, under these conditions of GIRK channel block, the mean E_GABAA_ still showed a depolarizing shift from −81.0 ± 2.2 mV to −73.6 ± 3.7 mV following SKF97541 application, which was a similar shift to that observed in control cells (*p* = 0.24, *n* = 7, ANOVA followed by *post hoc* Dunnett's correction; Fig. 3*F*). A separate set of experiments revealed that E_GABAA_ was not affected by the activation of postsynaptic adenosine receptors, the most abundant being the metabotropic A1 receptor, which like the GABA_B_R, is G_i_/_0_-coupled and targets rectifying potassium channels (Takigawa and Alzheimer, 2002; Ciruela et al., 2010a). The mean E_GABAA_ under baseline conditions was −84.5 ± 0.9 mV and was not significantly different following activation with adenosine (100 μm) at −83.7 ± 0.9 mV (*p* = 0.3, *n* = 13, paired *t* test). These data support the conclusion that the effect of GABA_B_R activation upon E_GABAA_ is specific to a GABA_B_R protein complex but does not involve the activation of downstream potassium channels.

To confirm that the effect of GABA_B_R activation was via changes in [Cl^−^]_i_, CA3 pyramidal neurons in organotypic hippocampal slices were transfected with a FRET-based chloride reporter protein called “Cl-Sensor” (Markova et al., 2008; Fig. 3*G*). Although there was no change in chloride concentration in control neurons expressing the Cl-Sensor protein (*p* = 0.67, paired *t* test, *n* = 13; Fig. 3*H*), activation of GABA_B_Rs with SKF97541 (5 μm for 20 min) resulted in a significant increase in the [Cl^−^]_i_ (*p* = 0.03, paired *t* test, *n* = 7; Fig. 3*H*). The change in [Cl^−^]_i_ was significantly greater in the SKF97541-treated neurons than in the control neurons imaged over the same time period (*p* = 0.02, *n* = 7 and 13, respectively, *t* test; Fig. 3*I*). Together, these data show that activation of the GABA_B_R can lead to a change in intracellular chloride regulation that is consistent with a decrease in KCC2 function.

### GABA_B_R activation can regulate KCC2 at the membrane

**Figure 4.**
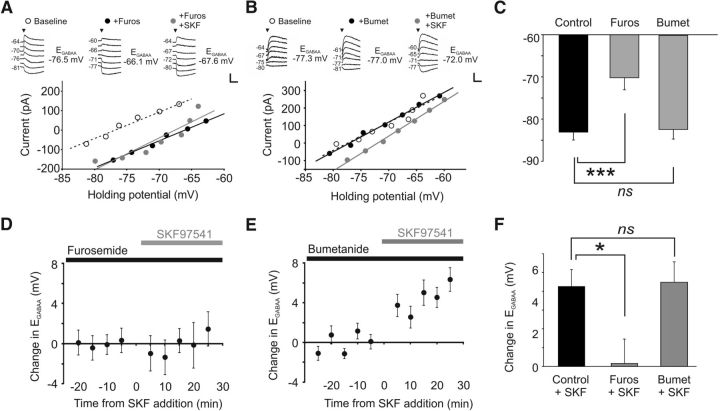
GABA_B_R activation downregulates the chloride transporter KCC2. ***A***, The GABA_B_R-induced shift in E_GABAA_ is occluded by blocking the activity of chloride transporter proteins. E_GABAA_ recorded in a CA3 pyramidal neuron in a rat organotypic hippocampal slice under baseline conditions (white symbols), following application of furosemide (1 mm, black symbols), and then after addition of SKF97541 (1 μm, gray symbols). Insets, Raw current traces recorded at different holding potentials. Calibration: 100 pA, 1 s. ***B***, The GABA_B_R-induced shift in E_GABAA_ is not prevented by blocking the chloride transporter NKCC1. E_GABAA_ recorded in a CA3 pyramidal neuron in a rat organotypic hippocampal slice under baseline conditions (white symbols), after bumetanide treatment (10 μm, black symbols), and then after the subsequent addition of SKF97541 (1 μm, gray symbols). Calibration: 100 pA, 1 s. ***C***, Compared with control cells (*n* = 9), E_GABAA_ was significantly more depolarized in neurons treated with furosemide (*n* = 12). ***p = 0.002 (ANOVA, with *post hoc* Dunnett's correction). Neurons treated with bumetanide (10–100 μm) had values comparable with control (*n* = 12). *p* = 0.97 (ANOVA, with *post hoc* Dunnett's correction). ***D***, The SKF97541-induced change in E_GABAA_ in a population of CA3 pyramidal neurons treated with furosemide (black bar) and plotted as a function of the time of SKF97541 application (gray bar; *n* = 12). ***E***, The SKF97541-induced change in E_GABAA_ in a population of CA3 pyramidal neurons treated with bumetanide (black bar), plotted as a function of the time of SKF97541 application (gray bar; *n* = 12). ***F***, The SKF97541-induced change in E_GABAA_ observed in control cells (*n* = 9) was blocked in furosemide-treated neurons (*n* = 12). *p = 0.039 (ANOVA, with *post hoc* Dunnett's correction). Bumetanide treatment did not affect the depolarizing shift in E_GABAA_ caused by GABA_B_R activation (*n* = 12). *p* = 0.98 (ANOVA, with *post hoc* Dunnett's correction).

To directly test the hypothesis that GABA_B_R activation modulates KCC2 function at the membrane, we performed a combination of electrophysiological recordings and biotinylation experiments. First, to establish that the GABA_B_R-mediated shift in E_GABAA_ occurs through a reduction in KCC2 function, cells were exposed to furosemide, which blocks KCC2 activity. CA3 pyramidal neurons exposed to furosemide (1 mm) exhibited a significantly more depolarized resting E_GABAA_ (−70.2 ± 2.9 mV, *n* = 12) than untreated control cells (−83.0 ± 1.9 mV, *n* = 9, *p* = 0.002, ANOVA followed by *post hoc* Dunnett's correction; Fig. 4*C*). The furosemide-induced shift in E_GABAA_ was evident within 5 min, highlighting that KCC2 functions to continuously maintain the hyperpolarized E_GABAA_ under these conditions. Importantly, application of the GABA_B_R agonist SKF97541 in the presence of furosemide failed to produce any further change in E_GABAA_ (−70.0 ± 2.3 mV) (Fig. 4*A*,*D*). Compared with control cells, the effect of SKF97541 upon E_GABAA_ was significantly attenuated in furosemide-treated cells (0.2 ± 1.3 mV, *p* = 0.039, ANOVA followed by *post hoc* Dunnett's correction; Fig. 4*F*).

As furosemide can block multiple cotransporter proteins, this experiment could not exclude a contribution by the sodium-potassium-chloride cotransporter protein, NKCC1, which has also been shown to contribute to [Cl^−^]_i_ regulation in hippocampal pyramidal neurons, particularly during development (Dzhala et al., 2005). We therefore examined the effect of GABA_B_R activation in the presence of bumetanide, a more selective blocker of NKCC1. The baseline E_GABAA_ after incubation with bumetanide was similar to untreated control cells, indicating that NKCC1 does not make a major contribution to the [Cl^−^]_i_ measured in these neurons (E_GABAA_ = −82.3 ± 2.3 mV, *n* = 12, *p* = 0.97, ANOVA followed by *post hoc* Dunnett's correction; Fig. 4*B*,*C*). Furthermore, bumetanide treatment did not prevent the depolarizing shift in E_GABAA_ following GABA_B_R activation. Upon SKF97541 application, the mean E_GABAA_ shifted to a new value of −77.8 ± 2.7 mV, a change that was indistinguishable to that seen in control cells (4.5 ± 1.1 mV, *p* = 0.98, ANOVA followed by *post hoc* Dunnett's correction; Fig. 4*E*,*F*). GABA_B_R activation led to an equivalent shift in E_GABAA_ in cells treated with both 10 μm bumetanide (baseline E_GABAA_ = −84.3 ± 3.2 mV, SKF97541-treated E_GABAA_ = −80.2 ± 4.2 mV, change = 4.1 ± 1.5 mV; *p* = 0.99, *n* = 6) or 100 μm bumetanide (baseline E_GABAA_ = −80.2 ± 3.3 mV, SKF97541-treated E_GABAA_ = −75.4 ± 3.3 mV, change = 4.8 ± 1.7 mV; *p* = 0.92, *n* = 6, ANOVA followed by *post hoc* Dunnett's correction). These data demonstrate that KCC2 is the mediator of the GABA_B_R-dependent effect upon E_GABAA_. If this is the case, one prediction is that other manipulations that downregulate KCC2 protein levels should attenuate the effects of GABA_B_R-mediated activation upon E_GABAA_. To test this idea, we maintained organotypic hippocampal brain slices in a zero Mg^2+^ ACSF for 3 h, which has been shown to cause a robust reduction in KCC2 levels and a depolarizing shift in E_GABAA_ (Puskarjov et al., 2012). Consistent with previous reports, the zero Mg^2+^ ACSF resulted in a depolarizing shift in E_GABAA_ (to −64.5 ± 4.9 mV). Importantly, activating GABA_B_Rs with SKF97541 in slices that had been treated in this manner caused no further change in E_GABAA_ (−66.6 ± 4.4 mV, *n* = 7, *p* = 0.28, *t* test). Together, these data confirm that KCC2 is the principle mediator of the GABA_B_R-dependent effect upon [Cl^−^]_i_.

**Figure 5.**
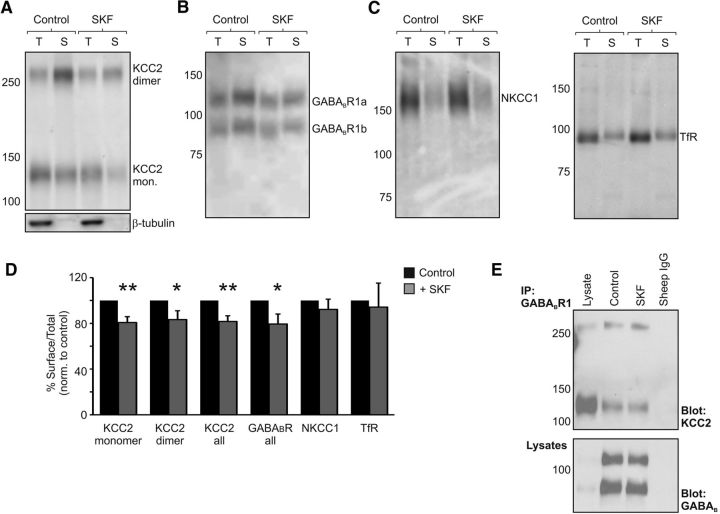
GABA_B_R activation leads to a reduction in KCC2 at the membrane surface. ***A***, GABA_B_R activation leads to a reduction in the fraction of both monomeric and dimeric forms of KCC2 at the membrane surface. Rat organotypic hippocampal slices (P7 + 7–14 DIV) were exposed to normal ACSF (Control) or ACSF containing SKF97541 for 20 min (SKF, 5 μm), and then biotinylated to label proteins at the cell surface. Cell homogenates of total protein (T) and streptavidin-purified cell surface proteins (S) were probed on Western blots with anti-KCC2 antibodies (top). Lack of β-tubulin staining in surface samples confirmed that the cell membranes remained intact (bottom). ***B***, SKF97541 treatment also led to a concomitant reduction in surface levels of GABA_B_R1 relative to controls. ***C***, Neither surface-bound NKCC1 nor the surface levels of the transferrin receptor changed following GABA_B_R activation. ***D***, Summary plot of the effect of GABA_B_R activation upon proteins at the membrane surface after 20 min 5 μm SKF97541 treatment. Surface expression was quantified as the ratio of surface to total protein, normalized to control slices that were examined in parallel. SKF97541 treatment resulted in a significant reduction in the surface ratio for KCC2 (monomer, **p = 0.002; dimer, *p = 0.048; all, **p = 0.003; *n* = 14, *t* test) and GABA_B_R1 (all, *p = 0.046, *n* = 9, *t* test). No effect was observed in the surface/total ratio for NKCC1 (*p* = 0.4, *n* = 13, *t* test) or the transferrin receptor (TfR; *p* = 0.79, *n* = 8, *t* test). ***E***, The association between KCC2 and GABA_B_R remains following SKF97541 treatment. Rat organotypic hippocampal slices were solubilized and subjected to coimmunoprecipitation analysis using GABA_B_R1 as the precipitating antibody. The amount of KCC2 in GABA_B_R1 complexes (top), normalized by the amount of GABA_B_R1 protein precipitated (bottom), did not change following SKF97541 treatment (*p* = 0.44, *n* = 3, *t* test).

To investigate whether the GABA_B_R-mediated decrease in KCC2 function involved regulation of the transporter protein at the cell membrane, we used biotinylation methods to isolate surface KCC2 from rat organotypic hippocampal slices. This approach has been used widely as a way to quantify changes in the plasmalemmal level of chloride transporter proteins in neuronal tissue (Rivera et al., 2002; Thomas-Crussells et al., 2003; Lee et al., 2007; Zhao et al., 2008). Having isolated surface KCC2 protein, we used quantitative Western blot analysis to express the amount of surface KCC2 protein as a ratio of the total KCC2 protein (surface/total KCC2; see Materials and Methods). Experiments revealed that, after 20 min of SKF97541 (5 μm) application, there was a reduction in KCC2 levels at the cell surface compared with control slices that were run in parallel but not exposed to the GABA_B_R agonist (Fig. 5*A*,*D*). Monomeric and dimeric forms of KCC2 were detected at the cell surface, and each showed a significant reduction with SKF97541 exposure, consistent with the hypothesis that GABA_B_R activation leads to a reduction in membrane bound KCC2 proteins. Monomeric KCC2 at the cell surface was reduced to 80.7 ± 5.1% of control levels (*p* = 0.002, *n* = 14, *t* test), whereas dimeric KCC2 was reduced to 83.3 ± 7.7% of control levels (*p* = 0.048, *n* = 14, *t* test; Fig. 5*A*,*D*). The effect was specific to KCC2 as surface levels of the transferrin receptor (94.2 ± 21%; *p* = 0.79, *n* = 8, *t* test) and NKCC1 (92.2 ± 8.9%; *p* = 0.4, *n* = 13, *t* test) were not statistically different between control and SKF97541-treated slices (Fig. 5*C*,*D*).

The effects upon surface KCC2 did not appear to involve degradation as the total amount of KCC2 protein was not different between control and SKF97541-treated slices. When examined after the same period of SKF97541 application (5 μm for 20 min), the normalized levels of total KCC2 monomer were 104.0 ± 7.1% of control and total KCC2 dimer was 103.7 ± 10.2% of control (*p* = 0.58 and *p* = 0.72, respectively; *n* = 14, *t* test). Further, when we repeated these experiments at the later time point of 30 min of SKF97541 application, we again found no evidence for degradation (the normalized levels of total KCC2 monomer were 102.6 ± 9.7% of control and total KCC2 dimer was 114.3 ± 18.5% of control, *p* = 0.79 and *p* = 0.46, respectively; *n* = 12, *t* test). Interestingly, at this later time point, an effect upon surface KCC2 was also not detectable (surface/total KCC2 monomer was 104.1 ± 8.3% of control and surface/total KCC2 dimer was 106.3 ± 9.9% of control, *p* = 0.63 and *p* = 0.54, respectively; *n* = 12, *t* test). These data suggest that GABA_B_R activation does not lead to KCC2 degradation but can rather affect the surface trafficking (endocytosis and recycling) of KCC2.

GABA_B_R dynamics at the cell surface can be affected upon receptor activation (Laffray et al., 2007; Grampp et al., 2008; Wilkins et al., 2008), suggesting that changes to surface trafficking of KCC2 could be associated with changes to surface GABA_B_Rs. Indeed, our electrophysiological recordings revealed that SKF97541-evoked currents tended to decrease in the continued presence of the agonist (a decrease of 10.1 ± 3.7%, from 88.5 ± 12.8 pA to 79.0 ± 11.1 pA, with 10 min of SKF97541 exposure; *n* = 13, *p* = 0.012, paired *t* test), indicating that there may be an agonist-dependent change in GABA_B_R signaling at the cell surface. To investigate this biochemically, we used our quantitative Western blot methods to examine GABA_B_R behavior after exposure to SKF97541 and found that, at the same time point that surface KCC2 is reduced (Fig. 5*A*,*D*), levels of surface GABA_B_Rs were also reduced. SKF97541 treatment (5 μm for 20 min) reduced GABA_B_R1 surface expression in the organotypic hippocampal slices to 79.4 ± 8.7% of control (*p* = 0.046, *n* = 9, *t* test; Fig. 5*B*,*D*). Meanwhile, coimmunoprecipitation experiments revealed that the amount of KCC2 pulled down in GABA_B_R1 complexes was not significantly different between SKF97541-treated and matched control slices (*p* = 0.44, *n* = 3, *t* test; Fig. 5*E*). To investigate whether the internalization of KCC2 via GABA_B_Rs could be reconstituted in the heterologous CHO cell system, we applied SKF97541 to CHO GABA_B_R1b/R2 transiently expressing FL-KCC2. In this system, there was no significant effect upon surface levels of either GABA_B_R1 (105.7 ± 3.4% of control; *p* = 0.12, *n* = 13, *t* test) or KCC2 (98.8 ± 6.1% of control; *p* = 0.85, *n* = 13, *t* test), suggesting that the mechanism may be sensitive to expression levels, post-translational modifications, or that intermediary proteins are involved in regulating surface expression in neurons.

### GABA_B_R regulation of KCC2 involves clathrin-mediated endocytosis

**Figure 6.**
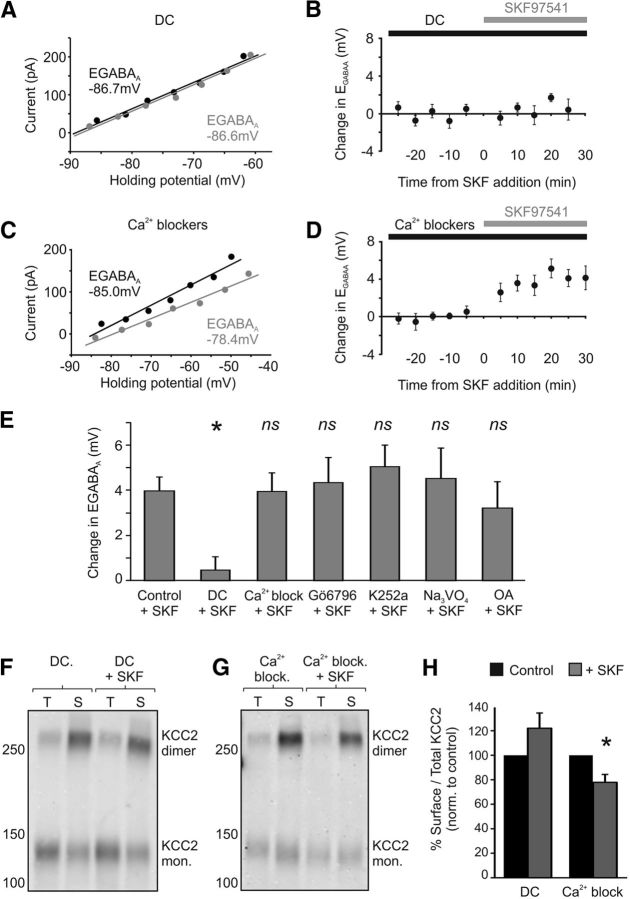
The GABA_B_R-effect upon KCC2 requires clathrin-dependent endocytosis. ***A***, Blocking clathrin-dependent endocytosis with DC prevents the depolarizing shift in E_GABAA_ following GABA_B_R activation. Example *I–V* plots are shown for a CA3 pyramidal cell in a rat organotypic hippocampal slice treated with DC (50 μm). E_GABAA_ at baseline (black data) is similar to that recorded after SKF97541 treatment (gray data). ***B***, The SKF97541-induced change in E_GABAA_ in a population of CA3 pyramidal neurons treated with DC (black bar) and plotted as a function of the time of SKF97541 application (gray bar; *n* = 8). ***C***, Blocking Ca^2+^ signaling via a combination of nimodipine (20 μm), thapsigargin (2 μm), and d-APV (100 μm) did not prevent the GABA_B_R-mediated shift in E_GABAA_. Same conventions as in ***A***. ***D***, The SKF97541-induced change in E_GABAA_ in a population of CA3 pyramidal neurons treated with Ca^2+^ channel blockers (black bar), plotted as a function of the time of SKF97541 application (gray bar; *n* = 9). ***E***, Summary plot of the effect of GABA_B_R activation in the presence of different inhibitors. Blocking clathrin-dependent endocytosis significantly reduced the SKF97541-dependent shift in E_GABAA_ (*n* = 8) observed in control cells (*n* = 11). *p = 0.03 (ANOVA followed by *post hoc* Dunnett's correction). In contrast, treating cells with a combination of Ca^2+^ channel blockers (*n* = 9) had no effect on the SKF97541-dependent shift in E_GABAA_. *p* = 0.99 (ANOVA followed by *post hoc* Dunnett's correction). Preincubation with the selective protein kinase C inhibitor, Gö6976 (1 μm, *n* = 9, *p* = 0.99); the general kinase blocker, K252a (100 nm, *n* = 6, *p* = 0.94); the tyrosine phosphatase inhibitor, Na_3_VO_4_ (1 mm, *n* = 6, *p* = 0.99); or the protein phosphatase 1 and 2 inhibitor, okadaic acid (1 μm, *n* = 6, *p* = 0.99) did not prevent a significant shift in E_GABAA_ following GABA_B_R activation (all ANOVA followed by *post hoc* Dunnett's correction). ***F***, Surface KCC2 levels are not altered when GABA_B_Rs are activated in the presence of an inhibitor of clathrin-dependent endocytosis. Rat organotypic hippocampal slices were pretreated with DC (50 μm) and exposed to SKF97541 (DC + SKF). Slices pretreated with DC but not exposed to SKF97541 (DC) were used as controls and run in parallel (see Materials and Methods). ***G***, Slices treated with a combination of Ca^2+^ blockers still showed reduced surface KCC2 levels following GABA_B_R activation. ***H***, Summary plot of the ratio of surface-to-total KCC2 protein, normalized to control values. Blocking clathrin-dependent endocytosis prevented the reduction in surface KCC2 following GABA_B_R activation (*n* = 7, *p* = 0.13, *t* test), whereas blocking Ca^2+^ signaling (*n* = 8) did not prevent the effect of GABA_B_R activation upon surface KCC2. *p = 0.011 (*t* test).

Both GABA_B_Rs and KCC2 undergo endocytosis via the clathrin-mediated endocytotic pathway (Grampp et al., 2007; Laffray et al., 2007; Vargas et al., 2008; Zhao et al., 2008). Indeed, clathrin-mediated endocytosis contributes to the constitutive membrane recycling of GABA_B_Rs, which can be accelerated by receptor activation (Grampp et al., 2007, 2008; Laffray et al., 2007). One possibility therefore is that the clathrin-mediated endocytotic pathway is important for the change in surface KCC2 that results from GABA_B_R activation. To test this, we used a selective blocker of the clathrin-mediated endocytotic pathway, dansylcadaverine (DC). Importantly, CA3 pyramidal neurons pretreated with DC (50 μm) failed to show a change in E_GABAA_ following GABA_B_R activation (Fig. 6*A*,*B*). E_GABAA_ shifted from a mean baseline of −82.6 ± 3.4 mV to −82.1 ± 3.3 mV following SKF97541 treatment, a shift of just 0.46 ± 0.6 mV (*n* = 8), which was significantly smaller than the change observed in control cells exposed to SKF97541 (*n* = 11, *p* = 0.03, ANOVA followed by *post hoc* Dunnett's correction; Fig. 6*E*). DC-treated neurons also failed to show a decrease in the amplitude of SKF97541-evoked currents in the continued presence of the agonist (a decrease of −0.9 ± 3.9%, from 36.3 ± 8.3 pA to 35.5 ± 7.8 pA, with 10 min of SKF97541 exposure; *n* = 8, *p* = 0.36, paired *t* test), suggesting no net change in GABA_B_R signaling at the cell surface. Finally, consistent with these electrophysiological recordings, the reduction in levels of surface KCC2 following GABA_B_R activation (5 μm SKF97541 for 20 min) was reduced when clathrin-mediated endocytosis was blocked with DC (50 μm; *n* = 7; *p* = 0.13; Fig. 6*F*,*H*). Disrupting the clathrin-mediated endocytotic pathway therefore occludes the GABA_B_R-mediated change in surface KCC2.

KCC2 function has also been reported to be modulated by Ca^2+^-dependent kinases, phosphatases, and proteases (Fiumelli et al., 2005; Lee et al., 2007, 2010; Wake et al., 2007; Xu et al., 2008; Watanabe et al., 2009; Puskarjov et al., 2012). To test whether the GABA_B_R-dependent modulation of KCC2 involves intracellular Ca^2+^ signaling, hippocampal slices were treated with a combination of Ca^2+^ channel blockers and intracellular Ca^2+^ store blockers (20 μm nimodipine, 100 μm d-APV and 2 μm thapsigargin). Under these conditions, GABA_B_R activation still resulted in a positive shift in E_GABAA_ that was indistinguishable from the shift in control slices (baseline E_GABAA_ = −78.8 ± 3.5 mV, SKF97541 treatment E_GABAA_ = 74.9 ± 4.3 mV, change = 3.9 ± 0.9 mV, *p* = 0.99, *n* = 9, ANOVA followed by *post hoc* Dunnett's correction; Fig. 6*C–E*). Blocking Ca^2+^ channels also failed to prevent the SKF97541-mediated (5 μm for 20 min) decrease in surface KCC2 measured by biotinylation (77.9 ± 6.4% *p* = 0.011, *n* = 8, *t* test; Fig. 6*G*,*H*). Consistent with these observations, the positive shift in E_GABAA_ was not prevented by inhibitors of calcium-dependent kinases. Pretreatment with Gö6796 (1 μm), a selective inhibitor of calcium-dependent protein kinase C, did not prevent the GABA_B_R-dependent shift in E_GABAA_ (baseline E_GABAA_ = −85.0 ± 3.0 mV, SKF97541 treatment E_GABAA_ = −80.7 ± 2.3 mV, change = 4.3 ± 1.1 mV, *p* = 0.99, *n* = 9, ANOVA followed by *post hoc* Dunnett's correction; Fig. 6*E*). K252a (100 nm), which inhibits protein kinase A and tyrosine kinases, also failed to block the GABA_B_R-dependent shift (baseline E_GABAA_ = −83.3 ± 3.7 mV, SKF97541 treatment E_GABAA_ = −78.2 ± 3.4 mV, change = 5.0 ± 1.0 mV, *p* = 0.94, *n* = 6, ANOVA followed by *post hoc* Dunnett's correction; Fig. 6*E*). Similarly, treatment with the tyrosine phosphatase inhibitor sodium orthovanadate (Na_3_VO_4_; 1 mm) did not prevent a positive shift in E_GABAA_ upon GABA_B_R activation (baseline E_GABAA_ = −71.5 ± 3.4 mV, SKF97541 treatment E_GABAA_ = −67.0 ± 3.7 mV, change = 4.5 ± 1.3 mV, *p* = 0.99, *n* = 6, ANOVA followed by *post hoc* Dunnett's correction; Fig. 6*E*). Treating cells with the phosphatase 1 and 2A inhibitor okadaic acid (1 μm), also failed to block the SKF97541-mediated shift in E_GABAA_ observed in control slices (baseline E_GABAA_ = −88.1 ± 0.7 mV, SKF97541 treatment E_GABAA_ = −84.9 ± 1.1 mV, change = 3.2 ± 1.2 mV, *p* = 0.99, *n* = 6, ANOVA followed by *post hoc* Dunnett's correction; Fig. 6*E*). Together, these data demonstrate that activation of the GABA_B_R leads to a decrease in the surface expression of KCC2, in a manner that is independent of calcium-dependent kinase and phosphatase activity, but is dependent upon clathrin-mediated endocytosis.

### Synaptically driven GABA_B_R activity affects intracellular chloride regulation

**Figure 7.**
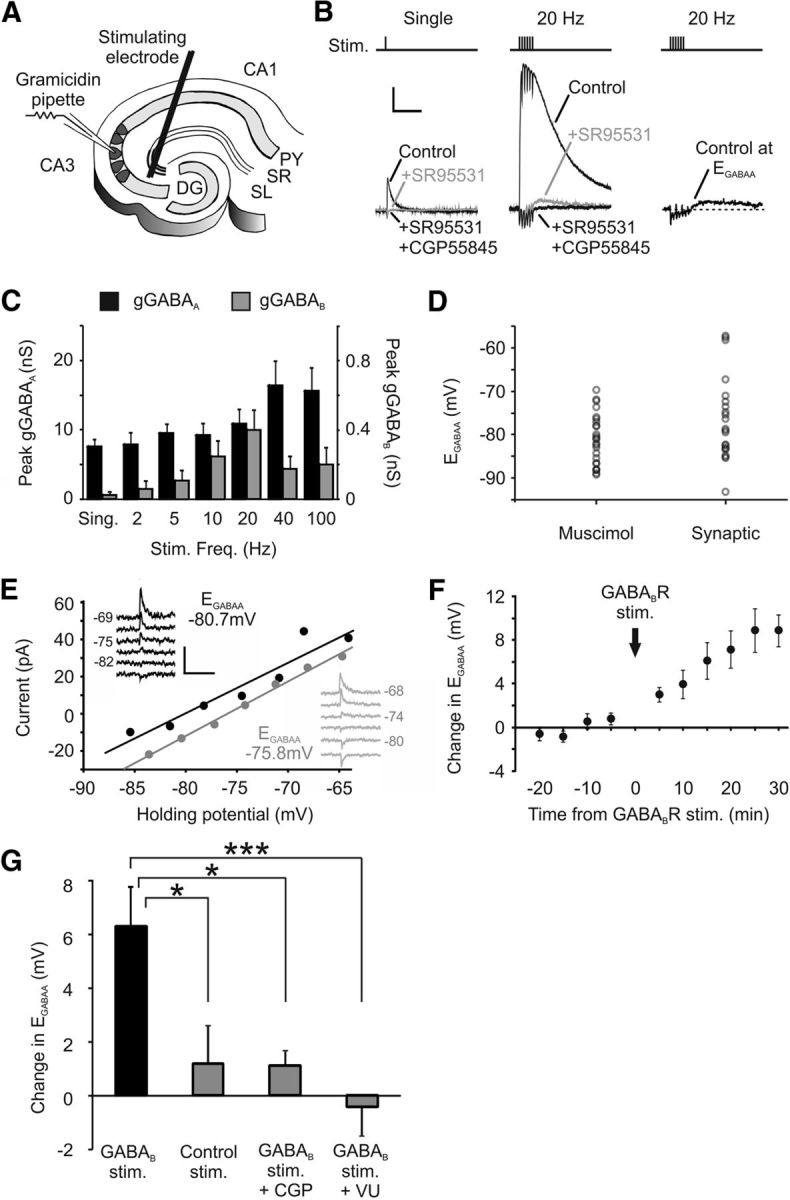
Synaptically driven GABA_B_R activation can shift E_GABAA_. ***A***, Diagram of the experimental setup for synaptically activating postsynaptic GABA_A_Rs and GABA_B_Rs. Presynaptic GABAergic interneurons were stimulated in rat organotypic hippocampal slices via a bipolar tungsten electrode positioned at the stratum radiatum/pyramidale border, 50–100 μm from the recorded cell. ***B***, Isolating GABA_A_R and GABA_B_R responses. Representative traces show monosynaptic GABAergic postsynaptic currents in a CA3 pyramidal neuron recorded in response to single presynaptic stimuli (left) or trains of 6 stimuli applied at 20 Hz (middle). GABA_A_R and GABA_B_R responses could be pharmacologically isolated by application of the selective GABA_A_R antagonist SR95531 (10 μm) and then the GABA_B_R antagonist CGP55845 (5 μm). GABA_B_R responses were not evoked by single stimuli but were evident for the multiple-stimuli condition. In the absence of these receptor blockers (right), the flux of chloride through GABA_A_Rs could be minimized by clamping the postsynaptic neuron close to its E_GABAA_. Calibration: 100 pA, 500 ms. ***C***, The amplitude of the postsynaptic GABA_B_R response is sensitive to presynaptic stimulus frequency. Whereas GABA_A_R conductances (gGABA_A_) were detected across the range of stimulus frequencies, GABA_B_R-mediated conductances (gGABA_B_) were largest for high-frequency stimuli of ∼20 Hz and were minimal at lower frequencies (*n* = 9). ***D***, Resting E_GABAA_ values measured by muscimol activation of the GABA_A_R (*n* = 25) and by synaptic activation of the GABA_A_R (*n* = 22). Synaptic E_GABAA_ exhibited a greater range of values and had a mean value of −76.7 ± 2.1 mV, compared with −81.1 ± 1.1 mV for the muscimol-evoked recordings (*p* = 0.06, *t* test). ***E***, Example GABA_A_R *I–V* plots for a CA3 pyramidal neuron before (black data) and after (gray data) delivering a conditioning protocol designed to strongly activate postsynaptic GABA_B_Rs (90 stimuli delivered as 15 bursts of 6 stimuli at 20 Hz, at 5 s intervals). Insets, Raw traces. Calibration: 50 pA, 1 s. ***F***, Change in E_GABAA_ in a population of CA3 pyramidal neurons (*n* = 6) following delivery of the GABA_B_R conditioning protocol (vertical arrow). ***G***, CA3 pyramidal neurons that underwent the GABA_B_R synaptic conditioning protocol (*n* = 6) showed a significantly larger positive shift in E_GABAA_ than neurons that experienced a control stimulation protocol (90 stimuli delivered at 1 Hz) designed to generate minimal GABA_B_R activation (*n* = 6, *p = 0.017, ANOVA followed by *post hoc* Dunnett's correction). The change in E_GABAA_ induced by the GABA_B_R synaptic conditioning protocol was also prevented by blocking GABA_B_Rs with the selective antagonist CGP55845 (*n* = 5, *p = 0.022) or by blocking KCC2 activity with VU0240551 (25 μm; *n* = 7, ***p = 0.001.

To investigate whether this mechanism could be recruited under physiological conditions, we examined whether the GABA_B_R-mediated effect upon KCC2 occurs at inhibitory synaptic connections. Presynaptic GABAergic interneurons in organotypic hippocampal slices were stimulated via a bipolar electrode placed at the border of the stratum pyramidale and stratum radiatum, 50–100 μm from the recorded pyramidal cell (Fig. 7*A*). This enabled us to evoke monosynaptic GABA_A_R responses and to measure synaptic E_GABAA_. Baseline synaptic E_GABAA_ was similar to muscimol-evoked E_GABAA_, with a mean value of −76.7 ± 2.1 mV (*n* = 22; Fig. 7*D*). We next examined whether the GABA_B_R-mediated effect upon E_GABAA_ could be elicited via synaptic activation of GABA_B_Rs. GABA_B_Rs are located predominantly extrasynaptically in hippocampal pyramidal cells and are thought to be activated under conditions of strong GABA release, such as occur during periods of high-frequency presynaptic firing (Scanziani, 2000). Consistent with this, a single presynaptic stimulus generated a pure GABA_A_R response in CA3 pyramidal neurons, which was entirely blocked by SR95531 (10 μm; Fig. 7*B*). In contrast, high-frequency trains of stimuli (e.g., 6 stimuli at 20 Hz) produced a postsynaptic response that was comprised of a large GABA_A_R conductance and a smaller GABA_B_R-mediated conductance that could be blocked by CGP55845 (5 μm; Fig. 7*B*). By varying presynaptic stimulation conditions, it was observed that the optimal presynaptic frequency for activating a GABA_B_R response was close to 20 Hz (Fig. 7*C*).

Having established the stimulation parameters for isolating the GABA_A_R response and for evoking robust GABA_B_R responses, we asked whether synaptically driven GABA_B_R activation could induce an activity-dependent shift in E_GABAA_. Using gramicidin perforated patch recordings, baseline E_GABAA_ was first determined by using single presynaptic stimuli to elicit a postsynaptic GABA_A_R response at different holding potentials (Fig. 7*E*). A synaptic stimulation protocol was then administered, which had been shown to elicit strong GABA_B_R activation and consisted of bursts of 6 stimuli at a frequency of 20 Hz, repeated every 5 s for a period of 75 s (GABA_B_R synaptic conditioning protocol; see Materials and Methods). To avoid loading the cells with chloride during these stimulation trains, the holding potential of the recorded cell was clamped at E_GABAA_, so that there was minimum flux of chloride through the GABA_A_R (Fig. 7*B*). After the GABA_B_R conditioning protocol, synaptic E_GABAA_ was then remeasured as before using single presynaptic stimuli. These experiments revealed that the GABA_B_R synaptic conditioning protocol caused a robust depolarizing shift in E_GABAA_ (Fig. 7*E*,*F*). Across a population of cells, the mean E_GABAA_ shifted from a baseline value of −73.3 ± 3.4 mV to −67.2 ± 4.6 mV when recorded 15 min after synaptic GABA_B_R stimulation, which represented a change in E_GABAA_ of 6.1 ± 1.7 mV (*p* = 0.014, *n* = 6, paired *t* test; Fig. 7*G*). A temporal analysis of the data showed that the shift in E_GABAA_ was evident 10 min following synaptic stimulation of the GABA_B_R (E_GABAA_ = −69.4 ± 4.1 mV, change = 4.0 ± 1.3 mV, *p* = 0.027, *n* = 6, paired *t* test) and was still detected at 30 min after stimulation, the longest population data point that we were able to record (E_GABAA_ = −64.5 ± 4.2 mV, change = 8.9 ± 1.4 mV, *p* = 0.002, *n* = 6, paired *t* test; Fig. 7*G*).

To establish that this effect was dependent upon GABA_B_R activation, we first confirmed that a control stimulation protocol that generated minimal GABA_B_R activation (90 stimuli delivered at 1 Hz) did not elicit a change in E_GABAA_. The baseline E_GABAA_ was −77.1 ± 4.1 mV; and after delivering the control stimulation protocol, E_GABAA_ was −75.9 ± 3.0 mV, which was a significantly smaller change in E_GABAA_ (1.2 ± 1.4 mV) than observed after the GABA_B_R conditioning protocol (*p* = 0.017, *n* = 6, ANOVA followed by *post hoc* Dunnett's correction; Fig. 7*G*). Then we confirmed that blocking GABA_B_Rs with a competitive antagonist (5 μm CGP55845) was able to significantly attenuate the shift in E_GABAA_ caused by the GABA_B_R synaptic conditioning protocol. Indeed, in these experiments, the baseline E_GABAA_ was −79.3 ± 1.2 mV; and after delivering the GABA_B_R synaptic conditioning protocol, the E_GABAA_ was −78.2 ± 1.7 mV. This was a change of 1.1 ± 0.5 mV, which did not represent a significant depolarizing shift in E_GABAA_ (1.1 ± 0.5 mV, *n* = 5; *t* test; *p* = 0.11) and was significantly smaller than the E_GABAA_ change observed without the GABA_B_R antagonist (*p* = 0.022, *n* = 5, ANOVA followed by *post hoc* Dunnett's correction; Fig. 7*G*).

Finally, blocking KCC2 with the selective antagonist VU0240551 (25 μm) (Delpire et al., 2009; Ivakine et al., 2013) also reduced any change in E_GABAA_ following GABA_B_R stimulation with the GABA_B_R conditioning protocol (baseline E_GABAA_ = −72.1 ± 3.6 mV, poststimulation E_GABAA_ = −72.6 ± 2.6 mV, change = −0.5 ± 1.1 mV, *p* = 0.001, *n* = 7, ANOVA followed by *post hoc* Dunnett's correction; Fig. 7*G*). These experiments demonstrate that the GABA_B_R-mediated effect upon E_GABAA_ via KCC2 is not only elicited by exogenous agonist activation of the GABA_B_R but can also be elicited by synaptically evoked GABA release.

## Discussion

By forming signaling complexes through specific interactions with other proteins, G-protein-coupled receptors convert extracellular signals into diverse neuronal responses. In the case of GABA_B_Rs, this includes G-proteins that are required for their “classic” signaling, but also interactions with auxiliary proteins that modulate the kinetics of receptor signaling (Schwenk et al., 2010), desensitization (Pontier et al., 2006), subunit dimerization (Couve et al., 2001), and regulate the localization of the receptor or other proteins within cells (White et al., 2000; Boyer et al., 2009). Here we have identified a novel association between the GABA_B_R and the potassium-chloride cotransporter KCC2. This association was discovered in an unbiased screen for proteins present within GABA_B_R complexes at the neuronal membrane, was confirmed by biochemical experiments in hippocampal brain slices and heterologous cells, and was shown to be mediated via the transmembrane region of KCC2. Agonist activation of the GABA_B_R elicits signaling events at the neuronal membrane via G-protein-coupled complexes. We observed that GABA_B_R activation led to a rapid and sustained change in the ionic driving force for the chloride-permeable GABA_A_R, consistent with a decrease in KCC2 function. Electrophysiological recordings and biotinylation assays confirmed that the effects were mediated via KCC2 and were associated with a change in the trafficking of KCC2 protein at the cell surface. A similar downregulation in KCC2 function could also be elicited by a synaptic conditioning protocol designed to strongly activate GABA_B_Rs. And while other signaling mechanisms may have been activated under our experimental conditions, the principle change in E_GABAA_ was unlikely to be mediated by alternative GABA receptors, such as the GABA_C_R, or by other signaling systems (Mahadevan and Woodin, 2016), because the effect of the GABA_B_R agonist and synaptically released GABA were both blocked by a selective GABA_B_R antagonist. These results are consistent with evidence that GABA_B_R activation modulates proteins with whom the receptor is physically associated (Ciruela et al., 2010b; Park et al., 2010).

In our recordings from CA3 pyramidal neurons, activation of the GABA_B_R by agonist or by synaptically released GABA resulted in an ∼5 mV positive shift in E_GABAA_, which is similar in amplitude to the shifts in E_GABAA_ following other activity-dependent changes to chloride transporter proteins (Woodin et al., 2003; Wang et al., 2006; Xu et al., 2008; Ormond and Woodin, 2009). Shifts in E_GABAA_ were evident within ∼10 min following GABA_B_R activation, which is also consistent with previous evidence that E_GABAA_ can be rapidly modulated within minutes (Woodin et al., 2003; Fiumelli et al., 2005; Wang et al., 2006; Balena and Woodin, 2008; Xu et al., 2008). Our longest recordings were unable to capture the reversal of the effects on E_GABAA_ and showed that they were evident for at least 30 min, which is again similar to previous studies that have examined the activity-dependent regulation of KCC2 function (Woodin et al., 2003; Fiumelli et al., 2005; Kitamura et al., 2008; Lee et al., 2011; Puskarjov et al., 2012; Zhou et al., 2012). Assuming E_GABAA_ reflects E_Cl_^−^ and that extracellular chloride remains constant, a 5 mV shift would equate to an increase in intracellular chloride of ∼1.2 mm (from 5.4 to 6.6 mm, according to the Nernst equation). Changes in E_GABAA_ over a narrow range (<5 mV) can have dramatic effects upon whether GABAergic inputs have an inhibitory or facilitating effect (Morita et al., 2006; Jedlicka et al., 2011) and E_GABAA_ changes of the same magnitude can cause significant changes in the degree of NMDA receptor activation and action potential firing frequency (Akerman and Cline, 2006; Saraga et al., 2008), which can be further influenced by the frequency and location of GABAergic inputs (Prescott et al., 2006; Jean-Xavier et al., 2007).

The GABA_B_R-mediated effect upon KCC2 appears to be distinct from previously described, activity-dependent mechanisms that regulate KCC2. Post-translational regulation of the transporter has been linked to calcium signaling events and associated enzymatic modifications. KCC2 function is associated with its phosphorylation state (Woodin et al., 2003; Fiumelli et al., 2005; Lee et al., 2007; Wake et al., 2007), and the transporter has been reported to turnover rapidly and as a function of the phosphorylation of specific sites within the C-terminal (Rivera et al., 2004; Lee et al., 2010). Recent work has revealed that the total pool of KCC2 is much more stable, but that degradation can be triggered by intracellular calcium, which activates calcium-dependent proteases that cleave the C-terminal of KCC2 (Puskarjov et al., 2012). In contrast to these mechanisms, the GABA_B_R-mediated effect upon KCC2 was not prevented by blocking calcium signaling processes, it was not affected by blockers of kinases and phosphatases implicated in the regulation of KCC2, and the total levels of KCC2 were not altered, suggesting that degradation pathways are not involved.

The stable physical association we observed between the GABA_B_R and KCC2, plus evidence that GABA_B_Rs can exhibit dynamic behavior at the membrane, offers a potential mechanism by which GABA_B_R activation could influence the surface stability and/or trafficking of the transporter protein. Previous work has provided differing results on the membrane dynamics of GABA_B_Rs. Some studies have reported that the receptor is stable at the cell surface, regardless of whether it is activated or not (Fairfax et al., 2004; Grampp et al., 2007). Other studies have provided evidence that GABA_B_Rs are mobile, being rapidly and constitutively internalized on a timescale of minutes via clathrin-dependent pathways, and in a manner that can be modulated by activation of the receptor (Laffray et al., 2007; Grampp et al., 2008; Wilkins et al., 2008). Our experiments in rat organotypic hippocampal slices revealed that GABA_B_R activation can result in a decrease in the surface expression of both GABA_B_R and KCC2 proteins. Such a reduction in surface GABA_B_R following receptor activation is consistent with previous observations in slice cultures (Laffray et al., 2007) but contrasts with studies in dissociated neuronal cultures (Fairfax et al., 2004; Vargas et al., 2008), suggesting that the experimental system (Vargas et al., 2008), or factors such as the dimerization state of the GABA_B_R (Laffray et al., 2007; Hannan et al., 2011), may be important. The effects we observed appeared to affect only a subset of the proteins (<25% decrease in both surface proteins) and were evident over a similar, but not identical, timescale to the downregulation in KCC2 function that we measured electrophysiologically. These differences in timescales of effect may reflect the sensitivities of the methods but could also indicate functional changes to KCC2 that result from being recycled to the membrane, perhaps due to changes in membrane domain, cellular location, and/or molecular interactions (Hartmann et al., 2009; Watanabe et al., 2009).

Internalized GABA_B_Rs are associated with the clathrin-binding adaptor protein-2 complex (Grampp et al., 2007), and disrupting clathrin-mediated endocytosis prevents internalization and recycling of GABA_B_Rs (Grampp et al., 2007; Laffray et al., 2007; Vargas et al., 2008). Similarly, KCC2 has been shown to bind to adaptor protein-2 in the brain and to undergo fast clathrin-mediated endocytosis (Zhao et al., 2008). Importantly, we found that blocking clathrin-mediated endocytosis prevented GABA_B_Rs from downregulating KCC2 function and expression at the neuronal membrane. Together, these data support a model in which active GABA_B_Rs can modulate the surface stability of KCC2 via a mechanism that involves clathrin-mediated endocytosis and which impacts the transporter's contribution to transmembrane chloride levels. It is worth noting that our data do not demonstrate a direct interaction between KCC2 and GABA_B_R; therefore, the potential for additional proteins to mediate the functional association in neurons should also be considered.

The fact that we observed a 20% reduction in surface KCC2 and a smaller GABA_B_R-mediated shift in E_GABAA_ than was produced by furosemide is consistent with the idea that different pools of KCC2 exist, which differ in terms of their localization, protein associations, and/or stability in the membrane. For instance, recent work has revealed that a pool of KCC2 is not localized at GABAergic synapses but rather at glutamatergic postsynaptic structures, where it functionally associates with kainate receptors and has been implicated in regulating glutamatergic transmission (Gauvain et al., 2011; Mahadevan et al., 2014; Chevy et al., 2015). Our experiments did not distinguish between protein complexes located in different subcellular compartments, such as the soma or dendrites. Future experiments could therefore explore whether the KCC2-GABA_B_R association varies as a function of cellular location or the membrane lipid environment (Hartmann et al., 2009; Watanabe et al., 2009). It will also be interesting to examine the longer-term consequences of manipulating the KCC2-GABA_B_R association, where the use of transgenic mouse lines is likely to be informative (Schuler et al., 2001; Vigot et al., 2006).

In conclusion, GABA_B_Rs are able to associate in a protein complex with the potassium-chloride cotransporter KCC2. Activation of the GABA_B_R can result in a decrease in KCC2 function, which requires the clathrin-mediated endocytosis pathway, regulates the transporter protein at the cell surface, and alters the driving force for chloride-permeable GABA_A_Rs. These findings reveal a novel “crosstalk” between the GABA receptor systems, which has important implications for the regulation of inhibitory synaptic transmission.
